# Human giardiasis in Serbia: asymptomatic *vs* symptomatic infection*

**DOI:** 10.1051/parasite/2011182197

**Published:** 2011-05-15

**Authors:** A. Nikolić, I. Klun, B. Bobić, V. Ivović, M. Vujanić, T. Živković, O. Djurković-Djaković

**Affiliations:** 1 Serbian Centre for Parasitic Zoonoses, Institute for Medical Research, University of Belgrade Serbia

**Keywords:** *Giardia*, humans, asymptomatic infection, symptomatic infection, Serbia, *Giardia*, homme, infection asymptomatique, infection symptomatique, Serbie

## Abstract

Despite the public health importance of giardiasis in all of Europe, reliable data on the incidence and prevalence in Western Balkan Countries (Serbia, Bosnia and Herzegovina, Croatia, Montenegro and FYR Macedonia) are scarce, and the relative contribution of waterborne and food-borne, or person-to-person and/or animalto- person, transmission of human giardiasis is not yet clear. To provide baseline data for the estimation of the public health risk caused by *Giardia*, we here review the information available on the epidemiological characteristics of asymptomatic and symptomatic human infection in Serbia. Although asymptomatic cases of *Giardia* represent a major proportion of the total cases of infection, high rates of *Giardia* infection were found in both asymptomatic and symptomatic populations. No waterborne outbreaks of giardiasis have been reported, and it thus seems that giardiasis mostly occurs sporadically in our milieu. Under such circumstances, control measures to reduce the high prevalence of giardiasis in Serbia have focused on person-to-person transmission, encouraging proper hygiene, but for more targeted intervention measures, studies to identify other risk factors for asymptomatic and symptomatic infections are needed.

## Abstract (fr)

Malgré l’importance en termes de santé publique de la giardiase humaine dans toute l’Europe, les données fiables sur son incidence et sa prévalence dans les pays de l’ouest des Balkans (Serbie, Bosnie-Herzégovine, Croatie, Monténégro et République de Macédoine) sont rares, et ses diverses modalités de transmission, via l’eau, les aliments, de l’animal à l’homme et de personne à personne ne sont pas encores claires. Afin d’apporter des données de base pour l’évaluation en santé publique du risque *Giardia*, l’article passe en revue les informations disponibles sur les caractéristiques épidémiologiques des infections humaines asymptomatiques et symptomatiques en Serbie. La giardiase asymptomatique concerne la majorité des cas d’infections, mais des taux élevés d’infections tant asymptomatiques que symptomatiques ont été trouvés dans des populations. Aucune épidémie d’origine aquatique n’ayant été observée, il semble que la giardiase survient surtout sporadiquement dans nos régions, c’est pourquoi les mesures visant à réduire la haute fréquence de la giardiase en Serbie se sont concentrées sur la transmission de personne à personne, avec la nécessité de promouvoir des mesures d’hygiène appropriées, d’interventions plus ciblées, d’études pour identifier d’autres facteurs de risque à la fois pour les infections asymptomatiques et symptomatiques.

Giardiasis, a common intestinal protozoan infection caused by *Giardia duodenalis* (syn. *G. intestinalis*, *G. lamblia*), is considered a neglected disease in both developed and developing countries (Savioli *et al.*, 2006). It is well documented that in developing countries, infections are associated with poor sanitary conditions, poor water quality and overcrowding (Younas *et al.*, 2008), whereas in developed countries cases are usually associated with international travel and immigration (Ekdahl & Andersson, 2005). Findings in stool specimens suggest a prevalence of 20-30 % in the developing countries, and of 2-5 % in the developed world (Ortega & Adam, 1997). In addition, giardiasis affects domestic and wild animals, and thus causes major public and veterinary health concerns worldwide.

Clinical presentations of giardiasis vary from asymptomatic carriage to acute diarrhoea as well as chronic disease, and at present, *Giardia* is recognized as the most common parasitological cause of diarrhoea, with 280 million infections per year. Most epidemiological studies of giardiasis indicated waterborne, food-borne, and person-to-person (or, to a lesser extent, animalto- person) transmission, depending on the study population (Cacció *et al.*, 2005). Person-to-person transmission is well recognized in crowded populations and in environments where hygiene levels may be compromised such as day care centres or schools (Thompson, 2008). Giardiasis usually occurs sporadically, although outbreaks have been reported. Waterborne outbreaks of giardiasis have been described many times in developed countries including the USA (Daly *et al.*, 2010) and Europe (Nygård *et al.*, 2006). In developing countries, the major risk factors include low personal hygiene, poor sanitation and urban overcrowding.

Despite the pan-European public health importance of giardiasis (Hörman *et al.*, 2004; Sagebiel *et al.*, 2009), data on the national incidence and prevalence in Western Balkan Countries (WBC), including Serbia, Bosnia and Herzegovina, Croatia, Montenegro and FYR Macedonia, are scarce. Moreover, waterborne outbreaks of giardiasis have not been reported in any of these countries, while the relative contribution of person-to-person, animal-to-person, waterborne and food-borne transmission to sporadic human giardiasis is still unknown. This article reviews the available data on the epidemiological characteristics of asymptomatic and symptomatic human infection in Serbia, to define the importance and risk for public health caused by *Giardia*.

## Asymptomatic Infection

**Fig. 1.**
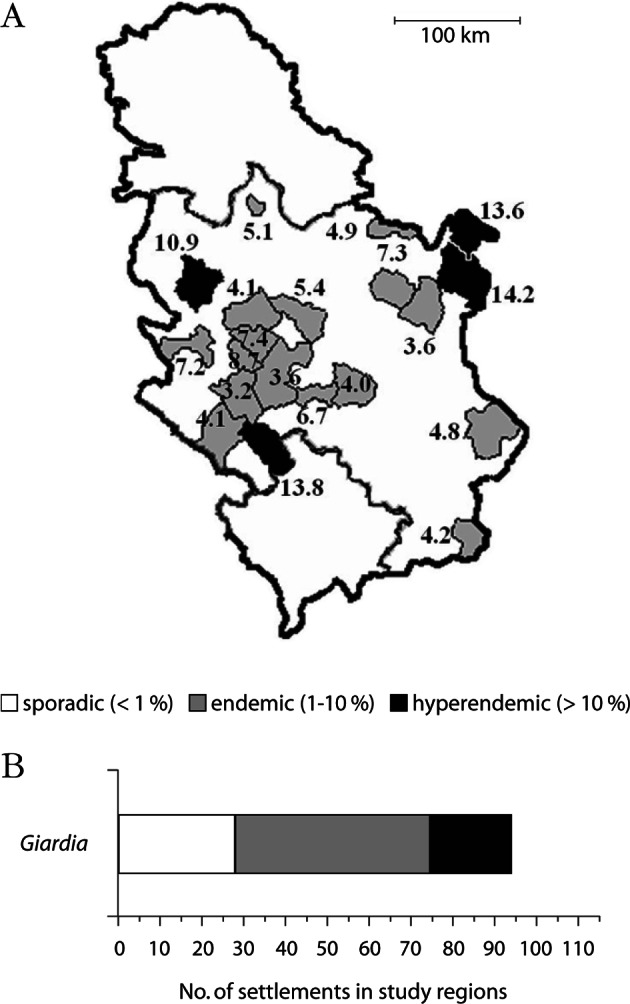
Asymptomatic human *Giardia* infection in Central Serbia: (A) map with prevalence (%) in 20 study regions and (B) distribution of prevalence in 115 settlements throughout Central Serbia.

To determine the epidemiological characteristics of asymptomatic *Giardia* infection in Serbia, several prevalence studies have been performed between 1985 and 2005 (Nikolić *et al.*, 2009). These studies involved a total of 6,645 asymptomatic schoolchildren, 7-11 years of age, representing 10 % of the total age-matched population (N = 69,232), from 115 settlements within 20 regions (Belgrade, Kragujevac, LuČani, Bor, Žagubica, Sjenica, Novi Pazar, Valjevo, Aleksandrovac, Pirot, Bosilegrad, Ivanjica, Golubac, Užice, Kladovo, Negotin, Kraljevo, Gornji Milanovac, Kruševac and ČaČak) throughout central Serbia. The results are presented in Fig. 1A. The study population, like in many others, comprised schoolchildren because they are an age group the most exposed to intestinal parasites, and are generally accessible. The results showed *Giardia* infection in all examined regions of Serbia, with an overall prevalence of 6.1 %, and highly significant (p = 0.001) differences among regions (3.2-14.2 %). According to the World Health Organization criteria (WHO, 1987) by which *Giardia* infection is considered sporadic at a prevalence below 1 %, endemic between 1-10 % and hyperendemic above 10 %, even 16 regions were endemic and four hyperendemic for giardiasis. These findings are important as understanding the spatial distribution may help to develop programmes for giardiasis control that combine chemotherapy (Nikolić *et al.*, 1995) and preventive measures.

Compared to other studies, the 6 % prevalence in Serbia is much higher than those reported in European countries such as Poland – 0.7 % (Bitkowska *et al.*, 2004) and UK – 1.3 % (Davies *et al.*, 2009), but lower than in Albania – 11.2 % (Spinelli *et al.*, 2006) and Turkey – 18.1 % (Ak *et al.*, 2006). There are no published data for giardiasis for other WBC, which obviously implies lack of awareness of this infection in the Balkans. High as it may seem, even the 6 % prevalence established in our studies is likely to be an underestimate, since the excretion of *Giardia* cysts is known to be intermittent, and examination of a single stool sample identifies no more than 75-85 % of all actually infected (Naik *et al.*, 1978; Hiatt *et al.*, 1995). However, the nature of epidemiological field studies does not allow for the examination of more than one stool sample (by several methods) and such a diagnostic approach has been widely accepted (rev. *in* Nikolić *et al.*, 1998).

Of the 115 settlements examined, only 21 were *Giardia*-free (Fig. 1B). The prevalence rates in the 94 *Giardia*-positive settlements varied from as low as 1.2 %, to a maximum of 22.7 %. Thus, infection with *Giardia* ranged from sporadic in 28, to endemic in 47 and hyperendemic in even 19 settlements. In the capital city of Belgrade, a highly urban setting, the detected prevalence of 5.1 % (Nikolić *et al.*, 1998) seems much higher compared to, for instance, the 1.6 % prevalence in asymptomatic individuals in Melbourne, Australia (Hellard *et al.*, 2000). Moreover, of all intestinal parasites detected in Serbia (*Entamoeba histolytica*, *E. hartmanni*, *E. coli*, *Iodamoeba bütschlii*, *Hymenolepis nana*, *Enterobius vermicularis*, *Ascaris lumbricoides*, *Trichuris trichiura*), *Giardia* was the single one, which was more prevalent (although insignificantly), in urban environments than in rural areas (7.0 % *vs* 6.5 %, respectively) (Nikolić et al., 1996). These findings support the significance of overcrowding in highly urban areas and living in close contact with infected persons for the spread of *Giardia*, and indicate that person-to-person transmission is an important means of spread of the infection in the community. Low median income was a risk factor for giardiasis in Serbia (Nikolić et al., 2002), which is in accordance with reports from other countries (Quihui *et al.*, 2006; Ostan *et al.*, 2007). The influence of income on infection may be associated with differences in human behaviour and living and environmental conditions that increase the risk of infection (Wilkinson *et al.*, 1992).

The differences in the rates of giardiasis in different settings in Serbia (urban *vs* rural, developed *vs* developing regions) indicate that the nature of giardiasis transmission depends on the population analyzed. Hence, control strategies of giardiasis should be based on epidemiological information for each particular population.

## Symptomatic Infection

National giardiasis surveillance should provide data to assess the epidemiological characteristics and estimate the disease burden by monitoring demographic parameters (sex, age), seasonality, and geographic variation (Furness *et al.*, 2000). While in countries such as USA and Canada human giardiasis is a notifiable disease, among members of the European Union reporting varies from voluntary in ones to mandatory in other member states. In Serbia, reporting is mandatory, and we here summarize the national giardiasis surveillance data as reported from January 2005 through December 2008 by the Institute of Public Health of Serbia (Health Statistical Yearbook, 2005, 2006, 2007 and 2008), and analyze the epidemiological trends.

**Fig. 2.**
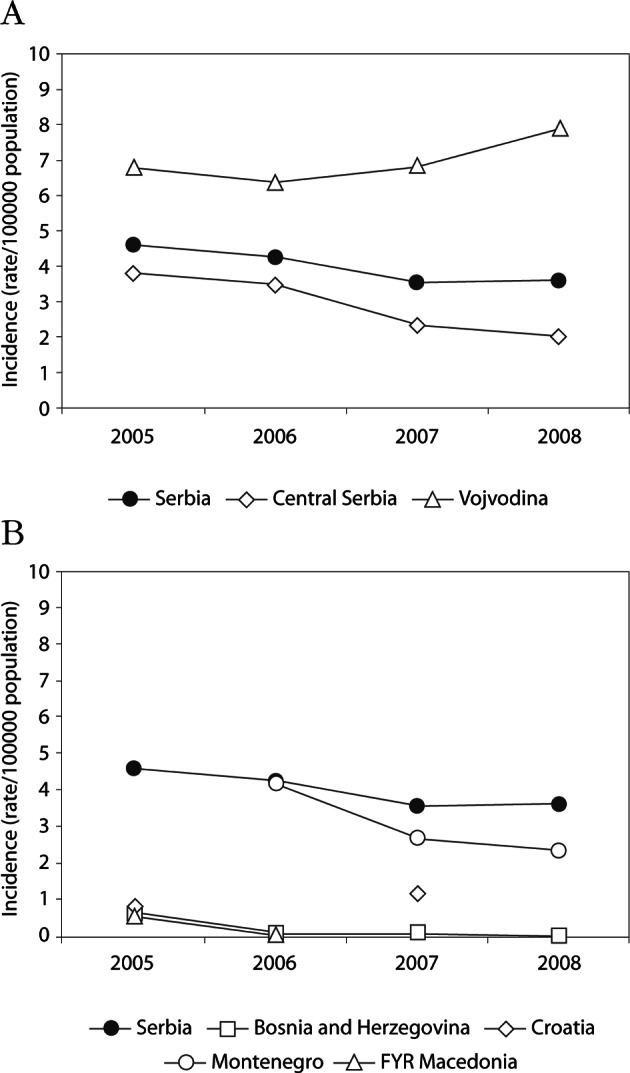
Changes in incidence of symptomatic human *Giardia* infections in Serbia *vs* (A) Vojvodina and Central Serbia and (B) other Western Balkan Countries (Bosnia and Herzegovina, Croatia, Montenegro and FYR Macedonia) between 2005 and 2008.

A total of 1,193 symptomatic cases were reported over this 4-year period. The number of cases has somewhat differed among the years, ranging from a high of 346 cases in 2005 (incidence 4.61 % per 100,000 population) to a low of 264 (incidence 3.55 %) in 2007, a decrease by 31 %, followed by a slight increase in 2008 (Fig. 2A). Whether the observed fluctuations reflect changes in reporting patterns or a real change in infection dynamics and disease caused by *Giardia* is unclear. Although giardiasis affects all age-groups, the number of reported cases was highest among adults aged 20-59 years (74.4 %), similar to findings in Germany where 83 % were age 20 or older (Espelage *et al.*, 2010). No sex-specific differences were observed *i.e.* the percentage of cases among males and females were 53 % and 47 %, respectively (p > 0.05).

Importantly, occurrence of symptomatic giardiasis in Serbia was associated with significant seasonality (seasonality χ^2^ = 41.576, p < 0.0001), with the highest number of cases reported between August and November (n = 470, 39.4 %) and the lowest number in the winter months (December – February) (n = 234, 19.6 %). Similar seasonal variation with most cases reported during late summer and early fall indicating that transmission occurred during summer has also been noted in the U.S. national surveillance data for giardiasis (Yoder & Beach, 2007; Yoder *et al.*, 2010). The peaks of incidence coincide with increased outdoor and water activities, and increased water consumption during hot weather.

Giardiasis is spread throughout Serbia. However, the northern province of Vojvodina consistently reported more cases annually per 100,000 population than Central Serbia (Fig. 2A). These data indicate that the diagnosis or transmission of giardiasis may be higher in the north. However, because differences in giardiasis surveillance systems among different areas can affect the capability to detect cases, whether this finding is of true biologic significance or is only the result of differences in case detection and/or reporting is difficult to determine.

**Fig. 3.**
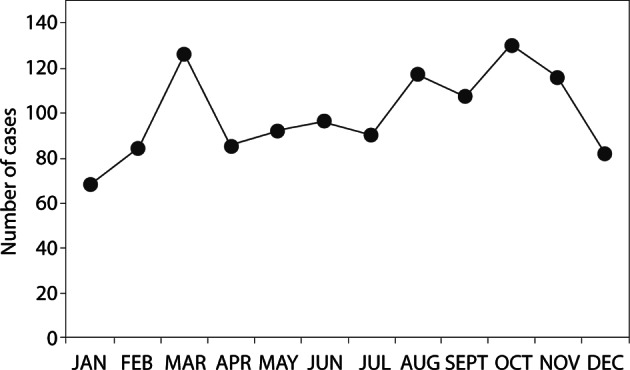
Total monthly counts of giardiasis for the four-year study period (2005-2008) in Serbia.

The incidence of *Giardia* infection varies not only among regions within Serbia, but between Serbia and other WBC as well (Fig. 2B). According to the data reported to the WHO, Serbia reported the greatest number of cases per 100,000 population for each of the four years of the reporting period compared to all other WBC (WHO/Europe, CISID, WHO/Europe, 2005-2008) (Fig. 3). However, this is likely to be at least in part due to insufficient reporting or monitoring systems (or both) among the WBC, as virtually no cases were reported in FYR Macedonia in 2006 and in Bosnia and Herzegovina in 2008, and some countries did not submit any giardiasis reports for some years (Croatia for 2006 and 2008, FYR Macedonia for 2007 and 2008, Montenegro for 2005). We believe that the reported giardiasis disease burden between 2005 and 2008 in the WBC is an underestimation, due to irregular reporting of Giardia infection on the national levels, as well as underreporting of diarrhoeal diseases in general.

## Conclusion

Human *Giardia* infection rates detected in Serbia over the last decades are high, both in symptomatic and asymptomatic populations. Although in Europe the prevalence of *Giardia* is lower in asymptomatic than in symptomatic populations (Hörman *et al.*, 2004), in Serbia asymptomatic cases of *Giardia* represent a major proportion of the total cases of infection and so giardiasis mostly occurs sporadically. However, the occurrence of outbreaks may not be ruled out. Person-to-person transmission of giardiasis is difficult to interrupt, particularly in groups such as schoolchildren. Thus, measures to reduce the high prevalence of giardiasis in Serbia should include adherence to appropriate infection control (*e.g.*, hand washing, chemotherapy of all infected individuals), while more targeted intervention measures require further studies to identify other risk factors for asymptomatic and symptomatic infections.
